# Cannabidiol prescription in clinical practice: an audit on the first 400 patients in New Zealand

**DOI:** 10.3399/bjgpopen20X101010

**Published:** 2020-02-05

**Authors:** Graham Gulbransen, William Xu, Bruce Arroll

**Affiliations:** 1 Private Practitioner, Cannabis Care NZ, West Care Specialist Centre, Auckland, New Zealand; 2 Medical Student, Department of General Practice and Primary Health Care, Faculty of Medical and Health Sciences, University of Auckland, Auckland, New Zealand; 3 Professor and Head of Department, Department of General Practice and Primary Health Care, Faculty of Medical and Health Sciences, University of Auckland, Auckland, New Zealand

**Keywords:** community care, therapy in mental health, prescribing, cannabidiol, cannabis, anti-anxiety agents, mental health, depression, chronic pain, analgesics, patient reported outcome measures

## Abstract

**Background:**

Cannabidiol (CBD) is the non-euphoriant component of cannabis. In 2017, the New Zealand Misuse of Drugs Regulations (1977) were amended, allowing doctors to prescribe CBD. Therapeutic benefit and tolerability of CBD remains unclear.

**Aim:**

To review the changes in self-reported quality of life measurements, drug tolerability, and dose-dependent relationships in patients prescribed CBD oil for various conditions at a single institution.

**Design & setting:**

An audit including all patients (*n* = 400) presenting to Cannabis Care, New Zealand, between 7 December 2017 and 7 December 2018 seeking CBD prescriptions

**Method:**

Indications for CBD use were recorded at baseline. Outcomes included EuroQol quality of life measures at baseline and after 3 weeks of use, patient-reported satisfaction, incidence of side effects, and patient-titrated dosage levels of CBD.

**Results:**

Four hundred patients were assessed for CBD and 397 received a prescription. Follow-up was completed on 253 patients (63.3%). Patients reported a mean increase of 13.6 points (*P*<0.001) on the EQ-VAS scale describing overall quality of health. Patients with non-cancer pain and mental-health symptoms achieved improvements to patient-reported pain and depression and anxiety symptoms (*P*<0.05). There were no major adverse effects. Positive side effects included improved sleep and appetite. No associations were found between CBD dose and patient-reported benefit.

**Conclusion:**

There may be analgesic and anxiolytic benefits of CBD in patients with non-cancer chronic pain and mental health conditions such as anxiety. CBD is well tolerated, making it safe to trial for non-cancer chronic pain, mental health, neurological, and cancer symptoms.

## How this fits in

CBD prescription in primary care was legalised in New Zealand in 2017. Previous preclinical trials have shown CBD to have anxiolytic and anti-inflammatory properties but there remains a paucity of studies investigating its therapeutic potential. In this quantitative observational study of the first 400 patients prescribed CBD in New Zealand, CBD was well tolerated amongst patients with a wide range of conditions and symptoms. Quality-of-life benefit was experienced to a greater degree in patients living with non-cancer chronic pain and anxiety-related mental-health conditions, and to a lesser degree in patients with cancer or neurological symptoms.

## Introduction

With the amendment of the New Zealand Misuse of Drugs Regulations 1977 in 2017, CBD has become a legal prescription medicine. The amendment recognises the right of New Zealand doctors to prescribe CBD products that contain no more of 2% of 9-Δ-tetrahydrocannabinol (9-Δ-THC) in the product.^1^

CBD and 9-Δ-THC are cannabinoids, active compounds found within the *Cannabis* genus of plants.^2^ While 9-Δ-THC is the main psychoactive component responsible for euphoria and the ‘high’ associated with marijuana, CBD is the non-euphoriant component.^2,3^

CBD is currently FDA-approved for the treatment of Dravet and Lennox-Gastaut syndrome, two childhood seizure disorders.^4^ Randomised controlled trials (RCTs) have shown that, when CBD is added to existing anti-epileptic medication in patients with these syndromes, seizure frequency decreases.^5,6^

However, CBD shows potential therapeutic use beyond this. Pre-clinical studies demonstrate that CBD has potential anti-inflammatory effects via inhibition of immune cell migration, which may be useful in chronic inflammatory conditions.^7^

Moreover, preclinical studies have demonstrated anxiolytic effects of CBD.^3,8–10^ Crippa *et al* found that in patients with generalised anxiety disorder given 400 mg of oral CBD, there was decreased cerebral blood flow to anxiety processing areas of the brain and a decrease in patient-reported anxiety when compared to placebo.^8^ CBD in one double-blinded placebo-controlled RCT decreased symptoms of social anxiety disorder patients and fear of public speaking.^10^ CBD may also reduce psychotic symptoms of schizophrenia.^11^

CBD appears to be safe for patients, with a recent phase I dosage trial showing purified CBD oil is well tolerated up to doses of 6000 mg.^12^ However, due to a paucity of clinical studies, prescribing guidelines are lacking.

The aim of this study was to conduct a clinical audit on the patient population referred to Cannabis Care (a primary care clinic in Auckland, New Zealand) for CBD oil. The authors explored the indications for prescribing CBD oil, patient quality of life, patient satisfaction, and self-titrated dosage levels.

## Method

The STROBE statement for reporting observational studies was followed.^13^

### Patients

Patients included in this audit were those who were prescribed CBD from 7 December 2017 to 7 December 2018. Patients included in this audit either were referred by their primary care provider or self-referred to the service. Patients were prescribed CBD oil (Tilray CBD100, Tilray, Nanaimo, BC, Canada) containing 100 mg CBD/mL in 25 mL bottles administered orally via a dropper. Bottles costed approximately USD300 each, which was self-funded as CBD is not on the New Zealand government-subsidised pharmaceutical schedule (PHARMAC).

### Outcomes

Patient sex, age, and details of medical condition were recorded at first consultation. Each participant was grouped into one of four broad clinical groups based on their presenting medical symptoms: non-cancer chronic pain symptoms, neurological symptoms, mental health-related symptoms, or cancer symptoms. When a patient presented with symptoms fitting multiple categories, the clinician assigned the patient to the category fitting their primary presenting complaint.

Patients completed an EQ-5D-5L questionnaire at baseline before taking CBD, and again after at least 3 weeks of using the medicine as part of routine clinical assessment. The EQ-5D-5L is a two-part tool consisting of the EQ-5D-5L descriptive system and the EQ Visual Analogue scale (EQ-VAS).^14^ The descriptive system measures five domains (mobility, self-care, usual activities, pain or discomfort, anxiety or depression) each with five levels of severity: no problems, slight problems, moderate problems, severe problems, and extreme problems. The EQ-VAS is a 20 cm vertical printed scale from 0 to 100 whereby the upper endpoint of the scale corresponds the ‘best health you can imagine’ and the lower corresponds to the ‘worst health you can imagine’. The patient rated their current overall health on this visual scale to yield a corresponding numerical score.

Patients also rated their satisfaction with their CBD use at follow-up. They rated their experience as ‘no benefit, good, very good, or excellent’ and reported any side effects. In addition, variations in patient dosage and the duration and frequency of CBD oil intake were recorded where possible.

### Statistical methods

The EQ-5D-5L data was measured on ordinal scales and hence considered as non-parametric data. The EQ-VAS scores were recorded on a continuous scale and treated as a parametric variable. Non-parametric data was presented as median (interquartile range [IQR]) and analysed for differences using the Wilcoxon rank sum test. Change in patient EQ-VAS scores was presented as mean (standard deviation [SD]) and analysed using one-way analysis of variance (ANOVA) to assess any differences between indication categories. Binary logistic regression was used to analyse potential dose-dependent responses, dose of CBD, and patient-reported benefit.

Categorical data such as the indications for CBD, patient satisfaction with CBD use, and side effects of use were presented as frequencies. A *P* value of ≤0.05 was taken as statistically significant. SPSS software (version 23.0) was used for statistical analysis.

## Results

A total of 400 patients presented to Cannabis Care from 7 December 2017 to 7 December 2018. Three patients did not receive a prescription based on a clinician decision that they would not benefit. Patients receiving CBD prescription consisted of 214 females (53.9%) and 183 males (46.1%), for a total of 397 individuals. The mean age of patients was 51.48 years (SD 19.1). Of those prescribed CBD, 61 patients (15.4%) fit more than one category of indication and were assigned to a group based on their primary condition.

Patient indication for CBD prescription is shown in Table 1. The non-cancer pain symptoms group included patients with fibromyalgia, osteoarthritis, rheumatoid arthritis, neuropathic pain, chronic non-specific pain, pain due to ulcerative colitis, and migraines. Cancer-related symptoms included pain, nausea, poor appetite, emotional distress, and adverse effects of radiotherapy and/or chemotherapy treatment. Mental health symptoms included anxiety disorders, depressive disorders, post-traumatic stress disorder, and insomnia. Neurological symptoms included Parkinson’s disease, multiple sclerosis, epilepsy, autism spectrum disorder with challenging behaviour, amyotrophic lateral sclerosis, multiple system atrophy, various neuropathies, and tremors.

**Table 1.** Baseline characteristics

Characteristic	Frequency, *n*	Proportion, %
Mean age, years (±SD)		51.48 (±19.1)	–
Sex	Male	183	46.1
	Female	214	53.9
Indication for CBD prescription	Non-cancer pain symptoms	181	45.6
	Mental health symptoms	64	16.1
	Neurological symptoms	60	15.1
	Cancer symptoms	92	23.2

Of the 397 patients initially prescribed the CBD oil, 253 (63.7%) were followed up either through a second appointment with the clinician or by phone. In total, 250 patients (63.0%) reported their satisfaction with CBD use, with three patients (0.8%) refusing to comment. Within these 250 patients, a subset of 110 patients (27.7%) completed before and after EQ-5D-5L questionnaires; 144 patients (36.3%) did not complete follow-up assessment, with 82 patients (20.7%) lost to follow-up and 62 patients (15.6%) choosing not to take the CBD. Reasons for patients not taking the CBD included death, financial barriers preventing purchase of the oil, severe illness, participation in a clinical trial, or consumption of alternative illicit cannabis products.

Median follow up duration for patients who completed their CBD prescription was 36 days (IQR 28–65).

### Outcomes of CBD treatment

Results from Wilcoxon rank sum tests for EQ-5D-5L domains found that patients experiencing non-cancer pain symptoms had a significant improvement of self-reported mobility scores (*P* = 0.02), ability to complete their usual activities (*P* = 0.007), self-reported pain (*P*<0.001), and self-reported anxiety or depression (*P* = 0.017). Patients with mental-health related symptoms experienced improvements to their ability to carry out their usual activities (*P* = 0.002), pain (*P* = 0.039), and anxiety or depression (*P* = 0.02). Patients with neurological symptoms experienced no statistically significant differences in any of the five domains. Patients with cancer symptoms experienced improvements in pain (*P* = 0.047). Complete results of EQ-5D-5L questionnaires are shown in Table 2.

**Table 2.** Baseline and follow-up EQ-5D-5L

**Indication for CBD prescription**	**Domain of EQ-5D-5L**	**Baseline EQ-5D-5L scores, median (IQR**)	**Follow-up EQ-5D-5L scores, median (IQR**)	***P value***
**Non-cancer pain symptoms** **(*n* = 53**)	Mobility	2.0 (1.0 to 3.0)	2.0 (1.0 to 3.0)	0.022
	Self-care	1.0 (1.0 to 2.0)	1.0 (1.0 to 2.0)	0.046
	Usual activities	3.0 (2.0 to 4.0)	2.0 (1.0 to 3.0)	0.007
	Pain/discomfort	3.5 (3.0 to 4.0)	3.0 (2.0 to 3.0)	<0.001
	Anxiety/depression	2.0 (1.0 to 3.0)	2.0 (1.0 to 2.0)	0.017
**Mental health symptoms** **(*n* = 21**)	Mobility	1.0 (1.0 to 1.0)	1.0 (1.0 to 1.0)	0.577
	Self-care	1.0 (1.0 to 1.75)	1.0 (1.0 to 1.0)	0.096
	Usual activities	3.0 (2.0 to 3.0)	1.0 (1.0 to 2.0)	0.002
	Pain/discomfort	2.0 (1.0 to 3.0)	1.0 (1.0 to 2.0)	0.039
	Anxiety/depression	4.0 (3.0 to 4.0)	2.0 (1.5 to 3.0)	0.002
**Neurological symptoms** **(*n* = 11**)	Mobility	1.0 (1.0 to 3.0)	1.5 (1.0 to 2.0)	0.317
	Self-care	1.0 (1.0 to 3.0)	1.5 (1.0 to 2.0)	0.317
	Usual activities	3.0 (1.75 to 4.0)	2.5 (1.75 to 3.25)	0.194
	Pain/discomfort	3.0 (1.5 to 3.5)	3.0 (1.5 to 3.0)	0.18
	Anxiety/depression	3.0 (2.0 to 3.0)	1.5 (1.0 to 3.0)	0.194
**Cancer symptoms** **(*n* = 24**)	Mobility	1.0 (1.0 to 2.0)	1.0 (1.0 to 2.0)	0.56
	Self-care	1.0 (1.0 to 2.0)	1.0 (1.0 to 1.0)	1
	Usual activities	2.0 (1.0 to 2.75)	2.0 (1.0 to 3.0)	1
	Pain/discomfort	3.0 (2.0 to 3.0)	2.0 (1.0 to 2.5)	0.047
	Anxiety/depression	2.0 (1.0 to 3.0)	1.0 (1.0 to 2.0)	0.11

Score of 1 = no problems, 2 = slight problems, 3= moderate problems, 4 = severe problems, 5 = extreme problems. *P* values are calculated from Wilcoxon rank sum tests. ‘Before’ scores taken at first consultation. ‘After’ scores taken after at least 3 weeks of cannabidiol intake.

CBD = cannabidiol. IQR = interquartile range.

Treatment with the CBD in all population groups showed an increase in the EQ-VAS score. There was an overall mean increase of EQ-VAS score of 13.6 points (*P*<0.001, 95% CI = 9.72 to 16.76). Overall, EQ-VAS scores represented an improvement in self-reported health. Results of one-way ANOVA tests showed that there were no significant differences between indication groups for the improvement of EQ-VAS scores.

Patient-reported satisfaction of CBD treatment found that 175 patients (70.0% of the 250 patients for whom there was available data, or 44.1% of the 397 patients prescribed the CBD), reported some level of satisfaction with CBD use (good, very good, or excellent). Seventy-five patients (30.0% of 250 patients, or 19.0% of 397 patients) reported no benefit from CBD use. There was no statistically significant relationship found between patient age or sex and patient-reported satisfaction.

### Side effects

Adverse effects such as sedation and vivid dreams were experienced by 25 out of 253 patients (9.9%). A worsening of a pre-existing condition was reported by 2 of 253 (0.8%) patients upon follow-up. Thirty-eight of followed-up patients (15.0%) reported positive side effects of CBD use, such as improved sleep or improved appetite. Side effects experienced by follow-up patients are summarised in Table 3.

**Table 3.** Side effect profile of the followed-up patients (*n* = 253)

**Side effect**	**Frequency, *n* (%)**
**Positive effects**
Improved sleep	31 (12.3)
Improved appetite	7 (2.8)
**Adverse effects**	
Sedation	5 (2.0)
Vivid dreams	5 (2.0)
Emotional disturbances eg, irritable, depressed, anxious	5 (2.0)
Disorientation	3 (1.2)
Sleeplessness	1 (0.4)
Nausea	1 (0.4)
Constipation	1 (0.4)
Diarrhoea	1 (0.4)
Headaches	1 (0.4)
Oral mucosa irritation	1 (0.4)
Hallucinations	1 (0.4)

### Dosage

Amongst those who completed the course of CBD, the dose per day ranged from 40mg/day to 300mg/day. However, dosage information was incomplete, existing for only 110 patients out of the 253 followed-up patients (43.5%). The clinician recommended dosing to at least 100 mg/day. Overall dosage between patients varied widely and was reported inconsistently by patients. Binary logistic regression analysis was conducted for the existing values and there was no significant association between dosage and patient-reported benefit from CBD (*P* = 0.145).

## Discussion

### Summary

CBD treatment improved self-reported quality of life measures for patients in the non-cancer pain and mental health-related symptom groups. There was no statistically significant improvement in those with cancer or neurological symptoms. Of those prescribed CBD, 44.1% (or 70.0% of the follow-up group) reported good to excellent benefit for relatively intractable conditions; 19.0% of those prescribed CBD (or 30.0% of the follow-up group) reported no benefit. CBD is well tolerated in most patients and may be of benefit to patients with various intractable chronic conditions.

### Strengths and limitations

The strength of this study is that it assesses effects of CBD on a large range of chronic medical conditions in a clinical context. Only three patients were excluded (due to extreme severity of their conditions).

There are several limitations to this present audit. There was a large loss to follow-up due to patients not attending follow-up and cost barriers. Hence, the results reported may not be fully representative of the entire patient population. Moreover, patients had to pay USD300 for 2500 mg of the CBD oil, USD150 for an initial consultation, and USD75 for follow-up. Most patients had refractory chronic pain and conditions resistant to conventional treatment uncommon in the wider population, increasing risk of selection bias. These patients are unlikely to be representative of a wider, more generalised cohort of patients. It is difficult to elucidate the effect of selection bias on these findings. The high cost barriers may augment the placebo effect of treatment,^15^ resulting in an overestimation of treatment effects. However, due to many patients having resistant symptoms, CBD may conversely prove more efficacious in patients with less severe symptoms.

The follow-up period was variable, resulting in interviews with some patients who had stopped taking CBD for a period of months. This may have confounded the patient’s recollection of the effects of CBD, likely causing an underestimation of the effect. Some follow-up was completed by the clinician instead of a non-clinical author. This increased the risk of expectation bias by the clinician, likely overestimating the effect. Considering these limitations, results should be interpreted with the appropriate caution.

### Comparison with existing literature

Numerous preclinical trials^9,10^ and neuro-imaging studies^8^ have demonstrated the anxiolytic effects of CBD. A recently published case-series in psychiatric patients found a benefit of CBD for anxiety and sleep,^16^ which is in agreement with the above findings.

On a pharmacological level, preclinical trials suggest CBD’s analgesic action is due to its effect on certain receptors: TRPV1 and a3 GlyRs, involved in nociceptive transmission. However, human studies are still inconclusive.^17–19^ The apparent benefit of CBD on pain reported by patients in this audit remains difficult to interpret. Current guidelines for the prescription of medicinal cannabis, including both 9-Δ-THC and CBD, recommend it as a third-line treatment for *‘* *neuropathic pain, palliative and end-of-life pain, chemotherapy-induced nausea and vomiting, and spasticity due to multiple sclerosis or spinal cord injury* *’*.^20^ However, RCTs investigating the use of CBD alone for the treatment of pain are lacking.

The ineffectiveness of the CBD oil for 75 of the 250 patients with available data (30.0%) may be due to lack of patient compliance and inadequate dosing. The expense of the pure CBD 100 mg/mL may have influenced patient attitudes towards this matter. Previous studies investigating CBD’s anxiolytic effect have shown a U-shaped dose curve with highest efficacy at 300 mg as a single-dose.^21^ This audit did not find a statistically significant dose-response, likely due to missing data and the aforementioned factors regarding financial cost.

CBD’s ineffectiveness in improving self-reported health in patients in the cancer and neurological groups may be due to the high heterogeneity of the clinical presentations within these groups. Additionally, patients within these groups had more severe disease progression compared to the mental health and non-cancer pain symptom groups, which may have limited efficacy.

Overall, CBD treatment was well tolerated with mild adverse effects, most commonly related to sedation. This is consistent with the findings of a phase I clinical trial showing the main side effects of CBD were related to the gastrointestinal and central nervous system.^12^ Patient-reported sleep benefits are likely related to these sedative effects. While cannabis (containing both 9-Δ-THC and CBD) has been indicated for use as an appetite stimulant in HIV-affected patients with cachexia,^22,23^ it remains unclear if CBD alone has significant appetite stimulating effects beyond placebo. Long-term side effects were not analysed in this current audit and future study is still needed to clarify chronic effects of CBD administration.^24^

### Implications for research and practice

Some urgency on this topic exists due to the increasing worldwide legalisation of cannabis and its related products. A focus should be placed on confirming the anxiolytic effects of CBD in clinical conditions. Future studies should investigate the effectiveness of full spectrum CBD oil with the retention of terpenes, the essential oils found in cannabis plants. Terpenes may enhance the effects of pure CBD because of a synergistic effect known as the entourage effect.

Overall, this audit demonstrated the potential benefit of CBD in treating anxiety and pain. The present study shows that it improves quality of life for a diverse range of patients. CBD in this population has been shown to be safe and well tolerated. However, despite potential biases of patients influenced by the high treatment cost, pure CBD is not effective for all. Benefit was more pronounced in patients who had conditions with less severe disease progression (such as mental health or non-cancer chronic pain conditions). Due to lack of a control group, high drop-out rate, and an extreme patient population, these study results should be interpreted with caution. Future studies should investigate effects of long-term CBD use, which could not be analysed in this present audit.
